# Demographic, clinical, and laboratory findings of mushroom-poisoned patients in Kermanshah province, west of Iran

**DOI:** 10.1186/s40360-022-00614-1

**Published:** 2022-09-26

**Authors:** Maryam Janatolmakan, Mohammad Rouhi Ganji, Touraj Ahmadi-Jouybari, Shahab Rezaeian, Mahnaz Ghowsi, Alireza Khatony

**Affiliations:** 1 Social Development and Health Promotion Research Center, Health Institute, Kermanshah University of Medical Sciences, Kermanshah, Iran; 2 Student Research Committee, Kermanshah University of Medical Sciences, Kermanshah, Iran; 3 Clinical Research Development Center of Imam Khomeini and Mohammad Kermanshahi and Farabi Hospitals, Kermanshah University of Medical Sciences, Kermanshah, Iran; 4 Infectious Diseases Research Center, Kermanshah University of Medical Sciences, Kermanshah, Iran; 5 Social Development and Health Promotion Research Center, Health Institute, Kermanshah University of Medical Sciences, Kermanshah, Iran; 6 Social Development and Health Promotion Research Center, Health Institute, Kermanshah University of Medical Sciences, Kermanshah, Iran; 7 Infectious Diseases Research Center, Kermanshah University of Medical Sciences, Kermanshah, Iran

**Keywords:** Epidemiology, Mushroom poisoning, Prevalence, Emergency

## Abstract

**Background:**

Mushroom poisoning can cause gastrointestinal, renal, and hepatic symptoms and even death. This descriptive study examined the demographic, clinical, and laboratory findings of patients with fungal poisoning, a type of fungus causing the poisoning, and the incidence and mortality rates of fungal poisoning in Kermanshah province, western Iran, from 2014 to 2018.

**Methods:**

The medical records of 193 patients with mushroom poisoning from 2014 to 2018 were evaluated. The liver and kidney function tests, electrolytes, abdominal and pelvic ultrasound, chest x-ray, coagulation tests, and coagulation factors (fibrinogen, prothrombin) were assessed. Data were collected from the medical records of patients admitted to the Poisoning Center of Imam Khomeini Hospital in Kermanshah, Iran using a researcher-made checklist. Data were analyzed by SPSS (version 16) using descriptive statistics, including mean, standard deviation, and frequency distribution tables. Trend analysis for proportion was done by chi-square statistics in STATA-14 software (ptrend command).

**Results:**

Of cases, ‌51.3% were male, 92.6% were city dwellers, 38.3% were aged 21–40 years, and 92.5% were poisoned during the spring. The fungus that caused poisoning was *Amanita virosa.* The gastrointestinal, nervous, and visual systems were the most common systems involved. The most common gastrointestinal symptoms included nausea and vomiting (72.0%) and abdominal pain (71.0%). Vertigo (11.9%) and headache (9.3%) were the most common neurological symptoms. The most common visual manifestation was blurred vision (7.8%). Of cases, 23.7% had metabolic acidosis. The increased alkaline phosphatase level was the most common liver disorder in 98.7% of the cases. Increased blood urea nitrogen and creatinine levels were also reported in 21.0% and 17.7% of the cases, respectively. The serum lactic dehydrogenase and creatine phosphokinase levels also increased in 99.3% and 30.2% of the patients, respectively. The mortality rate was 1.6% (n = 3).

**Conclusion:**

The fungal poisoning diagnosis should always be considered in young patients referred to the emergency department with gastrointestinal complaints, a history of consuming wild self-picked mushrooms, and high liver and kidney test values. Since most fungal poisonings occur in the spring, it is necessary to inform the community of the dangers of consuming self-picked wild mushrooms, especially in this season.

## Introduction

Of the 5000 mushroom species identified worldwide, about 3% are poisonous [1]. Eating small amounts of such poisonous mushrooms can cause liver, kidney, or even brain impairments and sometimes death [2–4]. The symptoms of mushroom poisoning depend on the toxin content of the mushroom and the age of the poisoned patient [5]. The most common cause of mushroom poisoning is the misidentification of a poisonous mushroom because of the similar color and shape of the poisonous species to the edible ones [6]. Clinical manifestations of mushroom poisoning are varied, but the most common ones include gastroenteritis, renal and hepatic injuries, neuropsychiatric complications, and hemolytic damage [7–9].

Mushroom poisoning is a critical health problem in many countries, including Bulgaria, the Czech Republic, China, Iran, Mexico, Italy, Hungary, Nepal, Japan, Poland, Romania, Russia, South Korea, Thailand, Turkey, and Ukraine [10]. The actual annual rate of death from mushroom poisoning in the world is unknown, but it is estimated to be at least 100 deaths, half of which occur in Europe [11]. A study in the United States (2020) reported an 8.8% death rate in 8953 mushroom poisoning cases [12]. In 2018 in Iran, following a string of mushroom poisoning events, more than 1200 cases were reported from 13 western and northwestern provinces, of whom 1.5% died [2].

In order to treat the poisoned patients, quick identification of the toxin type is of great importance, and obtaining a detailed history and examining the clinical signs and symptoms of the patient are the first-order treatment measures [13]. Since no comprehensive study on fungal poisoning has been conducted in Kermanshah-Iran, and there is no information on the demographic characteristics of poisoned people and their clinical and paraclinical findings, this study was conducted to determine the demographic characteristics and clinical and paraclinical findings of patients with fungal poisoning to detect the fungi species causing the poisoning, to determine the incidence rate, and also to determine the mortality rate during 2014–2018.

## Methods

### Study design

This retrospective descriptive study was conducted using the medical records of patients admitted to the Clinical Poisoning Center of Imam Khomeini Hospital in Kermanshah, Iran, from 2014 to 2018.

### **Study setting**

The study was conducted in Kermanshah city, the capital of Kermanshah province in Iran. Kermanshah province, located in Western Iran, has an area of ​​24,640 km^2^ and is the 17th largest and the 9th most populous province of Iran (1,900,000 people in the 2016 census). Kermanshah province is one of the tribal areas in the country, which has 14 cities and 84 villages [14, 15]. Different regions of Kermanshah province have very diverse climates, which provides a ground for the growth of toxic and non-toxic fungi. Therefore, the accidental eating of poisonous mushrooms leads to numerous poisoning cases in the province [14]. Imam Khomeini Hospital serves as a referral center for poisoning in Kermanshah province. The total number of patients referred to this hospital during the study period was 120,544, of whom 14,625 had referred due to various types of poisoning, and among these. Of these, 193 had mushroom poisoning.

### Sample and sampling method

A total of 193 hospitalized patients diagnosed with mushroom poisoning from March 21, 2014 to March 21, 2018 included the study population.

### Study instrument

The data collection tool was a researcher-made checklist that included items on gender, age, residence, occupation, poisoning route, season, and clinical findings.

### Data collection

After obtaining approval from the Ethics Committee of Kermanshah University of Medical Sciences, the author referred to Imam Khomeini Hospital to complete the checklists. For this purpose, all patients who were referred and hospitalized with a history of poisonous fungus consumption were included in the study. At Imam Khomeini Hospital, the diagnosis of fungal poisoning is mainly based on the patient’s history of wild mushroom ingestion, clinical picture of the disease, and paraclinical findings. However, to diagnose the type of mushroom causing the poisoning, samples of mushrooms consumed by patients were sent to the mycology laboratory of Kermanshah University of Medical Sciences.

Liver and kidney function tests, electrolytes, abdominal and pelvic ultrasound, chest x-ray, coagulation tests, and coagulation factors (fibrinogen and prothrombin) were assessed in all patients. It should be noted that the clinical symptoms studied in this study were related to the gastrointestinal, visual, nervous, integumentary, visual, respiratory, urinary, and cardiovascular systems.

Data were collected using electronic patient records. For this purpose, the researcher referred to the medical records unit and accessed the files of all patients using the keyword mushroom poisoning. If the files were deficient in demographic information, patients were contacted to complete their information as much as possible. It is noteworthy that the results of the first clinical evaluations and diagnostic tests were reported to the patients. The collected data were then fed into SPSS software for analysis.

### Data analysis

Data were analyzed by the Statistical Package for the Social Sciences (SPSS V.16.0; SPSS Inc., Chicago, IL, USA). Descriptive statistics, including mean, standard deviation, and frequency distribution tables were used to describe the data. Trend analysis for proportion was done by chi-square statistics in STATA-14 software (ptrend command).

### Ethical considerations

The ethics committee of Kermanshah University of Medical Sciences approved the study with the code KUMS.REC.1397.1053. The principle of confidentiality of patients’ information was observed.

## Results

A total of 193 patients were hospitalized in the study location from March 21, 2014 to March 21, 2018‌ due to the accidental eating of poisonous mushrooms. The mean age of the subjects was 43.1 ± 16.2 years, of whom 38.3% (n = 72) were within the age range of 21–40 years. Further, 51.3% (n = 97) were male, 75.1% (n = 145) were married, 78.6% (n =‌151) were unemployed, and 92.6% ‌(n‌=‌176) were urban dwellers. In addition, 92.5% (n=‌172) of the patients were poisoned in the spring from March 21 to June 21. In terms of the poisoning outcomes, the results showed that 75.3% (n‌=‌143) of the patients were discharged with complete recovery and 1.6% (n = 3) died (Table‌ 1).

In the present study, the type of poisonous fungus that caused poisoning was *Amanita virosa.*

The results showed that 44.6% of patients referred to the hospital 1–4 h after ingestion of poisonous mushrooms. The gastrointestinal, nervous, and visual systems were the most common systems involved. The most common gastrointestinal symptoms included nausea and vomiting (n = 139, 72.0%), abdominal pain (n = 137, 71.0%), and diarrhea (n = 65, 33.7%). Vertigo (n = 23, 11.9%), headache (n = 18, 9.3%), and lethargy (n = 13, 6.7%) were the most common neurological symptoms. The most common visual manifestations included blurred vision (n = 15, 7.8%) and tears (n = 2, 1.0%). The mean length of hospital stay was 4.1 ± 4.0 days (Table 2).

Of patients, 82.3% (n=‌121) had a systolic blood pressure of ≤ 120 mmHg. Diastolic blood pressure in 29.2% (n=‌50) of them was ≥ 71 mmHg. The mean body temperature of the patients was 37.01 ± 0.21 °C. Moreover, 23.7% (n = 45) of the patients had metabolic acidosis, 7.8% (n‌=‌14) had decreased platelet count. The results of prothrombin time (PT) and partial thromboplastin time (PTT) were ≥ 24 and ≥ 36 s in 5.1% (n = 9) and 27.5% (n‌= 49) of patients, respectively. Of patients, 16.9% (n = 30) had an international normalized ratio (INR) of ≥ 1.3 s. Alanine aminotransferase (ALT) and aspartate aminotransferase (AST) serum levels showed an increase in one-fourth of the patients. Moreover, 98.7% (n‌=‌178) of the patients had an alkaline phosphatase (ALKP) level of ≥ 121‌U/L. In addition, 25.9% (n‌=‌38) of the patients had a total bilirubin level of ≥ 1.1‌mg/dL, and 21.0% (n = 38) had a blood urea nitrogen (BUN) serum level of ≥ 41‌mg/dL. Furthermore, 17.7% (n = 32) of the patients had a creatinine (Cr) level of ≥ 1.3‌mg/dL, 99.3% (n=‌142) had a serum lactic dehydrogenase‌ (LDH) level of ≥ 191 U/L, and 30.2% (n = 49) had a serum creatine phosphokinase (CPK) level of ≥ 171 U/L (Table 3).

The results showed that the deceased patients included three men with a mean age of 50.6 ± 17.5 years. Two of them were self-employed and one was a farmer. One of these patients was addicted to drugs and alcohol. The mean length of stay in the intensive care unit (ICU) was 7.5 days among these patients. All three patients were poisoned by mountain mushrooms. The most common systems involved included gastrointestinal, cardiovascular, and nervous systems. The treatments received included gastric lavage, administration of charcoal, transfusion of blood products (pack cells and fresh frozen plasma), and drug therapy (pantoprazole, ondansetron, hydrocortisone, atropine, livorgol tablet, NAC, penicillin G, and lactulose syrup). Hemodialysis was also performed for one patient. All three patients were candidates for liver transplants. Patient death occurred 10 days, 5 days, and one and a half hours after admission to the hospital. In all three cases, the cause of death was cardiac arrest.

**Fig. 1:**
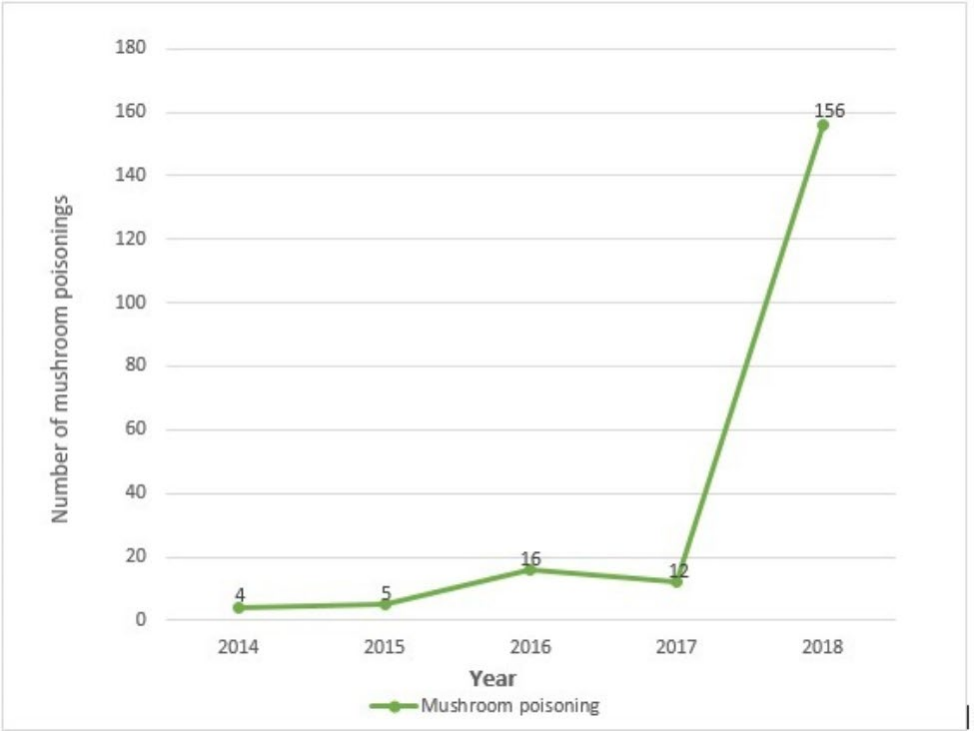
The trend of mushroom poisoning from 2014 to 2018

Trend analysis showed a significantly increasing trend in the incidence of mushroom poisoning from 2014 to 2018 (Fig. 1), which reached 53.86 per one million people in 2018 (Table 4).

**Table 1:** Demographic characteristics of patients with mushroom poisoning in Kermanshah, Iran during 2014–2018 (N = 193)

Variables		Number (%)
Sex	Female	92(48.7)
	Male	97(51.3)
Age (year)	≤ 20	17(9.0)
	21–40	72(38.3)
	41–60	68(36.2)
	≥ 61	31(16.5)
Marital status	Single	48(24.9)
	Married	145(75.1)
Discharge status	Recovered	143(75.3)
	Discharge with a doctor’s prescription	44(23.1)
	Expired	3 (1.6)
Season	Spring	172 (92.5)
	Summer	2(1.0)
	Fall	12(6.5)
	Winter	0(0.0)
Residence	Urban	176(92.6)
	Rural	14(7.4)
Occupation	Unemployed	151(78.6)
	Employee	41(21.4)
The interval between the consumption of mushrooms and the onset of symptoms and hospitalization (in hours)	≤ 6	140(73.7)
	7–12	39(20.5)
	13–24	10(5.3)
	25–48	1(0.5)
Duration of hospitalization (in days)	≤ 2	58(30.5)
	3–4	128(67.4)
	≥ 5	4(2.1)

**Table 2:** The most common manifestations of fungal poisoning in various body systems (N = 193)

Involved organs	Signs and symptoms	No (%)
Gastrointestinal (n = 158, 81.9%)	Nausea & vomiting	139 (72.0)
	Abdominal pain	137 (71.0)
	Diarrhea	65(33.7)
	Melena	6(3.1)
	Hematemesis	3(1.5)
Nervous (n = 55, 28.5%)	Vertigo	23(11.9)
	Headache	18(9.3)
	lethargy	13(6.7)
	Delirium	7(3.6)
	Sialorrhea	4(2.1)
	Loss of consciousness	1(0.5)
	Numbness	1(0.5)
	Facial paralysis	1(0.5)
Integumentary (n = 36, 18.6%)	Sweating	17(8.8)
	Fever and chills	9(4.7)
	Jaundice	7(3.6)
	Urticaria	2(1.0)
	Erythema	1(0.5)
Visual (n = 17, 8.8%)	Blurred vision	15(7.8)
	Tearing	2(1.0)
Respiratory (n = 4, 2.1%)	Dyspnea	4(2.1)
Urinary (n = 3, 1.5%)	Burning and frequent urination	2(1.0)
	Hematuria	1(0.5)
Cardiovascular (n = 2, 1.0%)	Chest pain	1(0.5)
	Tachycardia	1(0.5)

**Table 3:** Vital signs of patients with mushroom poisoning

Vital Signs	Normal ranges	Results	Number (%)
Diastole blood pressure (mmHg)	60–80	≤ 60	30(17.6)
Systolic blood pressure (mmHg)	90–120	121–140	23(15.6)
		141–160	2(1.4)
		≥ 161	1(0.7)
Respiratory rate (bpm^θ^)	12–20	13–22	186(98.4)
		≥ 23	2(1.1)
Pulse rate (bpm)	60–100	≥ 101	3(1.6)
Temperature (axillary; C°)	35.9–36.7	36.5–37	152(80.4)
		37.01-38	36(19.0)
		≥ 38.01	1(0.5)

Note: ^θ^ Beat per minute

**Table 4:** Annual distribution of mushroom poisoning in Kermanshah, Iran during 2014–2018 (N = 193)

Year	Total number of patients	Total number of different types of poisoning	Total number of mushroom poisoning	Population of Kermanshah province	Number of deaths due to mushroom poisoning	Incidence rate of mushroom poisoning per 1,000,000	Mortality rate per 10,000
2014	20,123	2845	4	2,382,986	0	1.68	0.25
2015	23,146	3150	5	2,502,135	0	2.00	
2016	27,036	2863	16	2,627,241	0	6.10	
2017	27,605	2713	12	2,758,603	1	4.35	
2018	22,634	3054	156	2,896,533	2	53.86	
*P*-value	-	-	-	-	-	0.001	-

Note: The denominator of incidence rate is the population of the province

## Discussion

The aim of this study was to investigate the epidemiological cases of mushroom poisoning in Kermanshah province during 2014–2018. The mean age of the studied patients was 43.1 ± 16.2 years. In a study in Turkey (2020), the mean age of patients with mushroom poisoning was 6.0 ± 3.2 years [16]. In studies conducted on mushroom poisoning in Iran, the mean age of patients ranged from 24.6 ± 16.7 to 30.5 ± 21.1 years [3, 5, 17]. In a study in China (2018), the age range of mushroom poisoned patients was 18–64 year [18]. In most of the studies, mushroom poisoning was reported in adults, which is consistent with the results of the current study. Due to the mountainous nature of Kermanshah city and the impassibility of surroundings, young people have less access to these areas, which can justify the high rate of poisoning in adults.

In the current study, there was no significant difference between genders in the frequency of poisoning, which was in line with the findings of the study by Khatir et al. (2020) in Iran [5]. In a study by Sattarzad Fathi et al. (2019) in Iran, the frequency of mushroom poisoning was higher in males [3]; however, the frequency of mushroom poisoning was higher in females in studies by Dadpour et al. (2017) in Iran and Ozdemir et al. (2019) in Turkey [17, 19]. Mushroom poisoning occurs in both genders; likewise, in the present study, the frequency of mushroom poisoning was the same in both genders.

In the current study, most mushroom poisoning cases occurred in the spring, which is consistent with the results of the study by Khatir et al. (2020)‌‌ in Iran [5]. But in studies by Sattarzad Fathi et al. ‌(2019) ‌‌in Iran, Jiang et al. (2018) in China, and Ozdemir et al. (2019) in Turkey, most cases of mushroom poisoning occurred in the winter, summer, and autumn, respectively [3, 18, 19]. Due to the mountainous and snowy nature of Kermanshah province, mushrooms mainly grow in the spring, which can justify the high frequency of such poisonings in this season.

Most cases in the current study were urban dwellers, while in a study in Turkey (2020), 57.9% of patients with mushroom poisoning were from rural areas [16]. Due to the presence of greenery around Kermanshah city, city dwellers go to nature for recreation, where the possibility of eating poisonous mushrooms is high. Rural people, however, are less likely to be poisoned by mushrooms since they have sufficient experience in identifying the poisonous types.

In the present study, about half of the patients were unemployed. In a study in Turkey (2020), most patients with mushroom poisoning were students and unemployed [16]. It seems that due to the greater leisure time available to unemployed individuals, they go to nature more frequently and, therefore, are at a higher risk for the accidental eating of poisonous mushrooms. On the other hand, due to the lack of job and steady income, unemployed people collect mushrooms as food for their families, which may be poisonous.

In the present study, the route of poisoning in all cases was accidental oral poisoning, which is in line with the findings of other studies [3, 6]. Due to the similar appearance of poisonous and edible mushrooms and the false belief that boiling poisonous mushrooms can make them safe, the possibility of accidental eating of such mushrooms is very high.

In the current study, the cause of poisoning was Amanita virosa in only 10% of cases and mountain fungi in 90%, in general. In studies conducted in China, the United States, and Iran, the type of mushroom consumed was different [6, 20–22]. In this regard, during the epidemic of fungal poisoning in Iran (2018), three types of fungi named Lepiota brunneioncarnata, Hypholoma fascicalare, and Coprinopsis atramentaria were introduced [2].

In the present study, the most common symptoms of fungal poisoning were gastrointestinal symptoms, which is consistent with the results of studies by Soltaninejad et al. (2019), Khatir et al. (2020), Badsar et al. (2013), and Schmutz et al. (2018) [2, 5, 23, 24]. However, in the studies of Trakulsrichai et al. (2017), Ma et al. (2017), and Wen et al. (2018), the most common manifestations of fungal poisoning were hepatic manifestations [21, 25, 26]. In the study of Vo et al. (2017), the gastrointestinal and hepatic systems had the highest rate of involvement [8]. Numerous factors play a role in the type of clinical manifestations and systems involved, including the type and amount of fungus consumed, the patient’s physical condition, and the underlying disease [23, 24, 25, 27].

In the current study, the vital signs of most patients were normal, which was in line with the results of studies by Karakoç et al.‌ (2020) and Anqi et al. (2017) [28, 29]. Li et al. (2019) reported an average blood pressure of 194/106 mmHg [30]. Changes in vital signs of patients with mushroom poisoning can be related to a variety of factors, including the patient’s age and the type and amount of mushroom eaten [25, 30].

In the present study, most cases had venous blood gas imbalance, and metabolic acidosis was the commonest disorder, while in studies by Karakoç (2020) and Karahan (2016) in Turkey, arterial blood gas values ​​were normal [28, 31]. Acid–base homeostasis is significantly affected by tissue and organ dysfunction, which can reflect poor organ functioning [32]. However, the different results can be attributed to different demographic characteristics of patients and the type and amount of mushrooms eaten.

In the current study, the PT, PTT, and INR values were normal in most of the cases, which is consistent with the results of the study by Khatir et al. (2020) in Iran [5]. In the study by Dadpour‌ et al. ‌(2017), the PT (24.6±‌24.6 s), PTT (33.8 ± 12.5 s), and INR (2.5 ± 3.6 s) values were abnormal [17]. Differences in results can be attributed to different demographic characteristics of the patients and the type and amount of mushrooms eaten.

In the current study, about one-fourth of patients had normal serum AST and ALT levels and abnormal total bilirubin level. ALK P levels showed an increase in most patients. However, in some studies, the results of liver function tests were normal [4, 5]. In the study by Dadpour et al. (2017), AST (434 ± 947 IU/L), ALT (534 ± 972 IU/L), and total bilirubin (4.0 ± 6.3 mg/dL) serum levels were abnormal [17]. In the study by Keller et al. (2018), the AST and ALT serum levels ​​were high (i e, ≥ 108 IU/L, ≥ 126 IU/L, respectively) [8]. In the study by Peng et al. (2018), 25% of the poisoned cases had severe hepatic impairments [7]. In studies by DeOlano et al. (2020), Karvellas et al. (2016), and Gul et al. (2020), the AST and ALT serum levels in most poisoned cases were abnormal [12, 16, 33]. Differences in results can be attributed to different demographic characteristics of the patients and the type and amount of mushrooms eaten.

In the present study, the results of kidney function tests, including BUN and Cr, were normal in most cases, while in the study by Hedman et al. (2017) in Sweden, the average serum levels of BUN and Cr were 31 ± 3.5 m‌mol/L and 1329 ± 133 µmol /L, respectively [4]. In studies by‌ Keller et al. (2018) and Ozdemir et al. (2019), the BUN and Cr levels showed an increase in patients with mushroom poisoning [8, 19]. Contradictory results can be attributed to different demographic characteristics of the patients and the type and amount of mushrooms eaten.

In the study period (from 2014 to 2018), the mortality rate of mushroom poisoning was 1.6% (n = 3). According to various studies, the mortality rate of mushroom poisoning in different countries ranges from 0 to 27.3% [2, 4–6, 9, 12, 17, 19]. Different factors, including the time elapsed between eating mushroom and referral to the emergency room, the type and amount of mushrooms eaten, and the quality of medical and nursing services, contribute to the mortality rate of mushroom poisoning [34, 35].

### Study limitations

This study faced several limitations. In 90% of patients, the fungus that caused poisoning was mountain fungus, which included various types of self-picked fungi that were not identified in the patients’ files. Since the current study was of a retrospective type, it was conducted based on patients ‘medical records, and some of the required information was not recorded in the patients’ files, which was collected by contacting the patients. In the present study, only the results of the first diagnostic tests and clinical evaluations were reported, and the results of subsequent times were not reported, which can be considered a limitation.

## Conclusion

The trend of mushroom poisoning has been increasing in recent years. To reduce the prevalence of this poisoning, increasing public awareness about the dangers of consuming self-picked wild mushrooms should be considered. Further, most cases of mushroom poisoning occurred in adults, urban dwellers, and unemployed subjects during the spring. Renal and hepatic impairments and ABG changes were the main clinical and paraclinical manifestations. Therefore, facing clients with renal and hepatic impairment symptoms as well as ABG changes, the possibility of mushroom poisoning should be considered. Similar studies in areas with a high frequency of mushroom poisoning are recommended.

## Data Availability

The data that support the findings of this study are available from Alireza Khatony, but restrictions apply to the availability of these data, which were used under license for the current study, and so are not publicly available. Data are, however, available from Alireza Khatony upon reasonable request.
